# All-*trans *retinoic acid inhibits KIT activity and induces apoptosis in gastrointestinal stromal tumor GIST-T1 cell line by affecting on the expression of survivin and Bax protein

**DOI:** 10.1186/1756-9966-29-165

**Published:** 2010-12-16

**Authors:** Hoang Thanh Chi, Bui Thi Kim Ly, Takahiro Taguchi, Toshiki Watanabe, Yuko Sato

**Affiliations:** 1 Division of Ultrafine Structure, Department of Pathology, Research Institute, National Center for Global Health and Medicine, Tokyo, Japan; 2 Department of Medical Genome Sciences, Graduate School of Frontier Sciences, the University of Tokyo, Tokyo, Japan; 3 Graduate School of Integrated Arts and Sciences, Doctoral Course, Kuroshio Science, Kochi University, Kochi-shi, Kochi-ken, Japan

## Abstract

**Background:**

Imatinib, a selective tyrosine kinase inhibitor, has been used as a standard first-line therapy for irresectable and metastasized gastrointestinal stromal tumor (GIST) patients. Unfortunately, most patients responding to imatinib will eventually exhibit imatinib-resistance, the cause of which is not fully understood. The serious clinical problem of imatinib-resistance demands alternative therapeutic strategy. This study was conducted to investigate the effect of all-*trans *retinoic acid (ATRA) on GIST cell lines.

**Methods:**

Cell proliferation was determined by trypan blue dye exclusion test. Western blot analysis was performed to test the expression of activated KIT, its downstream proteins, and apoptosis associated proteins. The cytotoxic interactions of imatinib with ATRA were evaluated using the isobologram of Steel and Peckham.

**Results and conclusion:**

In this work, for the first time we have demonstrated that ATRA affected on cell proliferation of GIST-T1 and GIST-882 cell line through inhibition of cell growth in a dose dependent manner and induced apoptosis. High dose of ATRA induced morphologic change in GIST-T1 cells, rounded-up cells, and activated the caspase-3 protein. In further examination, we found that the ATRA-induced apoptosis in GIST-T1 cells was accompanied by the down-regulated expression of survivin and up-regulated expression of Bax protein. Moreover, ATRA suppressed the activity of KIT protein in GIST-T1 cells and its downstream signal, AKT activity, but not MAPK activity. We also have demonstrated that combination of ATRA with imatinib showed additive effect by isobologram, suggesting that the combination of ATRA and imatinib may be a novel potential therapeutic option for GIST treatment. Furthermore, the scracht assay result suggested that ATRA was a potential reagent to prevent the invasion or metastasis of GIST cells.

## Background

Gastrointestinal stromal tumors (GISTs) are the most common mesenchymal neoplasms occurring throughout the entire region of the gastrointestinal tract and are considered to originate from intestitial cells of Cajal, the pacemaker cells of the gut [1]. The most likely causative molecular event in the vast majority of GISTs is a gain-of-function mutation of *KIT *or *PDGFRA *(platelet-derived growth factor receptor alpha) which activates these receptor tyrosine kinases (RTKs) by rendering them constitutively phosphorylated [2-4]. Thereafter, the downstream signaling pathways are activated promoting cell proliferation and/or survival.

To date, surgical resection seems to be the only treatment approach for GISTs with resulting in 5 year survival rates of 48-54% for resectable cases [5] while for irresectable or metastasized GIST cases, the median survival period was only 19 months and 5 year survival rate of 5-10% [6]. More recently, imatinib (Glivec, Gleevec; Novartis Pharma AG), a selective inhibitor of KIT, PDGFRA, ABL, as well as the other certain tyrosine kinases, has been used as a standard first-line therapy for irresectable and metastasized GISTs [7-11]. Clinical evidence supporting the indication of imatinib for GISTs was obtained from phase II/III trials in patients with irresectable GISTs [12]. Although imatinib has shown prominent effects to metastatic lesions of GIST, serious problems involved in imatinib-resistance have been reported recently [13,14]. The resistance develops after a median of about 2 years of treatment with imatinib [15]. Other KIT inhibitors such as sunitinib, PKC412 or BMS-354825 are reported to be effective in a subset of patients with imatinib-resistant GISTs. However, none of them have been proven to be effective to all the known imatinib-resistant mutations of *KIT *[16-18]. Therefore, development of novel KIT inhibitors or finding novel therapeutic strategy for GISTs is demanded.

Vitamin A (retinol) is a fat-soluble vitamin essential for the formation and maintenance of many body tissues, such as skin, bone, and vasculature, as well as for the promotion of good vision and immune function [19]. Vitamin A also plays a role in reproduction and in embryonic growth and development. Vitamin A is converted to more active compounds, such as retinoic acid, through which it exerts its multiple effects on embryonic development and organogenesis, tissue homeostasis, cell proliferation, differentiation, and apoptosis [20,21]. Retinol has six known biologically-active isoforms: all-*trans*, 11-*cis*, 13-*cis*, 9,13-di-*cis*, 9-*cis*, and 11,13-di-*cis *with all-*trans *being the predominant physiological form. Endogenous retinoids with biological activity include all-*trans *retinoic acid, 9-*cis *retinoic acid, 11-*cis *retinaldehyde, 3,4-didehydro retinoic acid [22].

The functions of retinoic acid regulating differentiation, proliferation and apoptosis are mediated by nuclear receptors, such as retinoic acid receptors (RARs) and retinoic × receptors (RXR) [23]. Although the mechanisms of retinoic acids on regulating differentiation, proliferation and apoptosis are not fully elucidated, it has been suggested that induction of differentiation and apoptosis by retinoic acids might contribute to treatment of cancers.

In this work, we studied the effect of ATRA on GIST cells in term of inhibition of cell proliferation, and induction of apoptosis. For the first time we have demonstrated that ATRA inhibited cell proliferation of GIST-T1 and GIST-882 cell line in a dose dependent manner and caused apoptosis. The apoptosis induced by ATRA may be regulated at least by down-regulated expression of survivin and up-regulated expression of Bax.

## Materials and methods

### Cell lines and culture conditions

The human GIST cell lines, GIST-T1 with 57-nucleotide (V570-Y578) in-flame deletion in *KIT *exon 11 [24], and GIST-882 cells with K642E mutation in exon 13 of *KIT *and the human normal diploid fibroblast cells (WI-38) (IFO 50075, Human Science Research Resource Bank, Osaka, Japan) were used in this study.

The cells were grown in Dulbecco's modified Eagle's medium (DMEM) with high glucose (Nakalai Tesque, Kyoto, Japan) supplemented with 10% fetal bovine serum (FBS) (JRH Biosciences, Lenexa, KS, USA), 100 IU/ml penicillin, and 0.1 mg/ml streptomycin (Nakalai Tesque) in a humidified incubator of 5% CO_2 _at 37°C.

### Reagents

Imatinib and all-*trans *retinoic acid were purchased from Sequoia Research Products (Oxford, UK) and WAKO Chemicals (Osaka, Japan), respectively. Both of them are dissolved in DMSO. The concentration of DMSO was kept under 0.1% throughout all the experiments to avoid its cytotoxicity.

### Cell proliferation assays

Cell proliferation was determined by trypan blue dye exclusion test. Cells were seeded in 6-well plates at a density of 1 × 10^5 ^cells/ml in the presence of different concentrations of ATRA or imatinib for 72 hours in humidified incubator of 5% CO_2 _at 37°C. After the treatment, the cells were washed twice with PBS without Ca^2+ ^and Mg^2+ ^[PBS(-)] to remove the medium. Then cells were dissociated with EDTA-trypsin solution. Ten micro liter of the cell suspension was mixed with 10 μl of 0.4% trypan blue, and alive cells were counted manually using a hemacytometer. Results were calculated as the percentage of the values measured when cells were grown in the absence of reagents.

### Western blot analysis

Cells were plated onto 10-cm dishes at a density of 1 × 10^5 ^cells/ml in the presence of 180 μM ATRA. After incubation for indicated durations, cells were collected by trypsinization and washed twice with PBS(-). Cell protein was extracted and western blot analysis was done as described previously [25]. The following antibodies ERK1 (sc-93), total Akt (sc-1618), anti-KIT antibody (cKIT-E1), survivin (sc-17779), anti-rabbit IgG-HRP (sc-2317), and anti-mouse IgG-HRP (sc-2031) were purchased from Santa Cruz Biotechnology (Santa Cruz, CA, USA). Anti-actin (A2066) was from Sigma-Aldrich. Phospho-p44/42 Map kinase (Thr202/Tyr204), phospho-Akt (Ser473), XIAP, caspase-3, phospho-c-Kit (tyr719) antibodies were from Cell Signaling Technology Japan (Tokyo, Japan). Anti-PARP antibody was from WAKO Chemicals (Osaka, Japan).

### Cell morphologic assessment

Cells were plated at a density of 1 × 10^5 ^cells/ml in the presence of different concentration of ATRA onto 6-well dishes. After 3-day treatment, cell morphology was observed under an inverted microscope.

### Wright-Giemsa staining

For fragmented nuclei and condensed chromatin assessment, cells at a density of 1 × 10^5 ^cells/ml were treated with 180 μM ATRA. After indicated durations, cells were harvested and fixed onto slides by using a cytospin (Shandon, Shandon Southern Products Ltd., Cheshire, UK). Cells then were stained with Wright-Giemsa solution. Morphology of cells was observed under an inverted microscope.

### DNA fragmentation assay

GIST-T1 cells were treated with or without 180 μM ATRA for different durations. Cells then were collected and total genomic DNA (gDNA) was extracted with a standard protocol. For DNA fragmentation assay, 10 μg gDNA of each sample was blotted and electrophoresed on 1.2% agarose gel. DNA fragmentation was detected under UV light.

### Scratch assay

GIST-T1 cells were seeded in 6-well plates with or without reagent. After 24-hour treatment, a line was scraped within confluent cells using the fine end of 10 μL pipette tip (time 0). After 24 hours, migration of GIST cells was observed under an inverted microscope.

### Assessment of cytotoxic effect of ATRA in combination with imatinib

The cytotoxic interactions of imatinib with ATRA were evaluated using the isobologram of Steel and Peckham [26]. The IC_50 _was defined as the concentration of reagent that produced 50% cell growth inhibition.

### Statistical analysis

All data were expressed as the mean ± standard deviation. Statistical analyses were done using Student's *t-test*, in which p < 0.05 was the minimum requirement for a statistically significant difference.

## Results

### Growth inhibitory effect of ATRA on GIST-T1 cells

**Figure 1:**
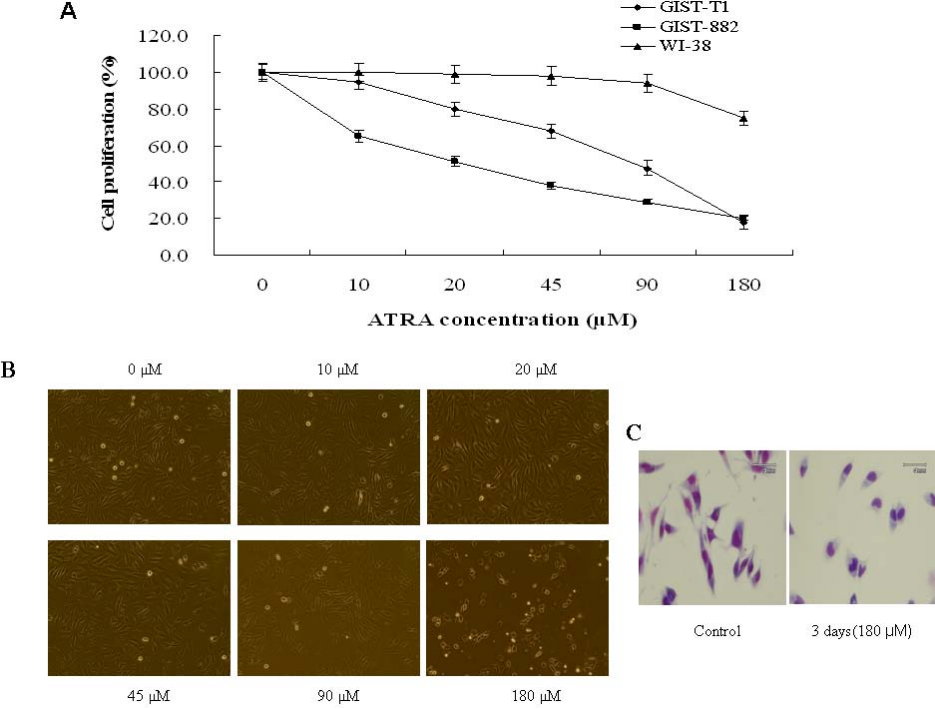
**Effect of ATRA on cell proliferation of GIST-T1, GIST-882 and human normal fibroblast WI-38 cells**. GIST-T1, GIST-882 and human normal fibroblast WI-38 cells at a density of 1 × 10^5 ^cells/ml were treated with different concentrations of ATRA dissolved in DMSO or with DMSO alone (0 μM ATRA as control) for 3 days. **Panel A **shows cell growth curve which represents the effect of different concentrations of ATRA. Results were calculated as the percentage of the control values. **Panel B **shows the effect of ATRA on adherence of GIST-T1 cells at various concentrations of ATRA. **Panel C **shows cell morphologic change of GIST-T1 cells after 3-day treatment with 180 μM ATRA.

ATRA treatment resulted in inhibition of cell proliferation of GIST-T1 and GIST-882 cells in a dose-dependent manner but showed nearly no effect on the human normal fibroblast WI-38 cell (Figure 1A). The adherence of GIST-T1 cells was much inhibited by ATRA-treatment in a dose-dependent manner (Figure 1B). In addition, ATRA treatment highly affected on morphology of GIST-T1 cells. ATRA-treated (180 μM, 3 days) GIST-T1 cells changed to rounded-up cells compared with the control cells (Figure 1C), suggesting that ATRA might cause inhibition of peripheral attachment in these cells. The effect of ATRA on morphological changes in GIST-882 cells was similar to GIST-T1 cells (data not shown).

### ATRA induced apoptosis in GIST-T1 cells

To confirm whether ATRA induces apoptosis in GIST-T1 cells, we further investigated apoptotic markers, nuclei shrinkage, DNA fragmentation and activation of caspase-3 in GIST-T1 cells after ATRA treatment.

**Figure 2:**
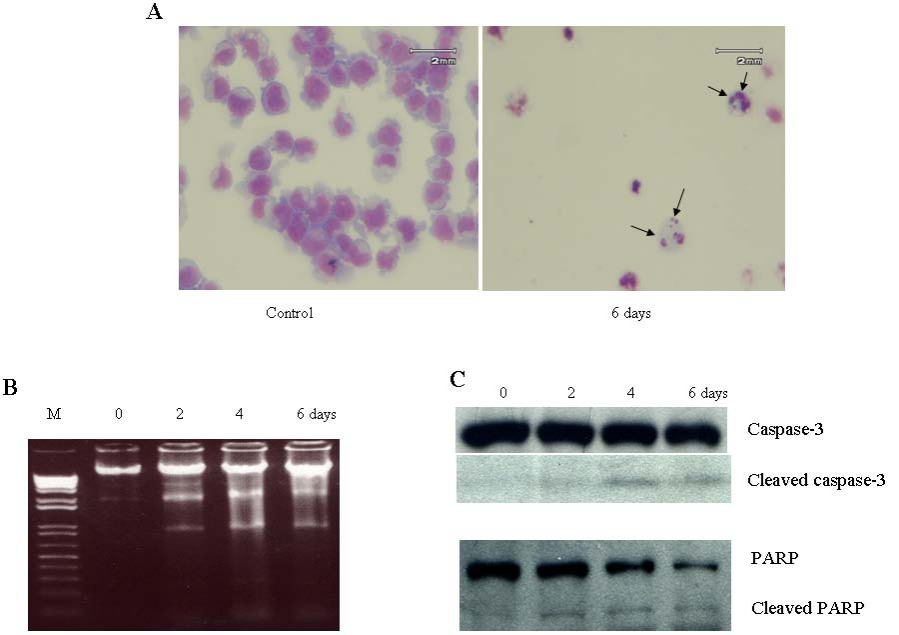
**ATRA induces apoptotic cell death in GIST-T1 cells**. **Panel A **shows the shrinkage and fragmentation of nuclei in GIST-T1 cells after 6-day treatment with 180 μM ATRA compared with the control cells. **Panel B **shows the result of DNA fragmentation after 2-, 4- or 6-day treatment with 180 μM ATRA. **Panel C **shows the presence of cleaved caspase-3 and cleaved PARP after 2-, 4- or 6-day treatment with 180 μM ATRA.

As mentioned above, ATRA not only induced the morphologic change (rounded-up cells) in GIST-T1 cells after 3-day treatment, but also induced detachment of the cells from the dishes after 6-day treatment (data not shown). To check whether detached cells show the features of apoptosis, cells were collected and fixed onto slides by using a cytospin before performing Wright-Giemsa staining. The result showed that detached cells showed shrunk and fragmented nuclei, the apoptotic features, compared with control cells (Figure 2A right), the fragmented nuclei were confirmed by DNA fragmentation assay (Figure 2B). As expected, DNA fragmentation was observed after 2-day treatment and increased in a time dependent manner.

Moreover, to clearly demonstrate that ATRA causes apoptosis in GIST-T1 cells, we assessed the molecular aspects of apoptosis, such as caspase-3, well recognized as a marker of apoptosis, and PARP, considered as a biochemical marker of necrosis when it is hyperactivated [27], by western blot. After 2-day treatment with 180 μM ATRA, cleaved caspase-3 and PARP were observed (Figure 2C). This result is consistent with the data of DNA fragmentation, demonstrating that ATRA induced apoptosis in GIST-T1 cells.

Overall, our results demonstrated that ATRA induced apoptotic cell death in GIST-T1 cells. The similar result was also confirmed in GIST-882 cells (data not shown).

### ATRA affected on expression of survivin, XIAP and Bax protein

**Figure 3:**
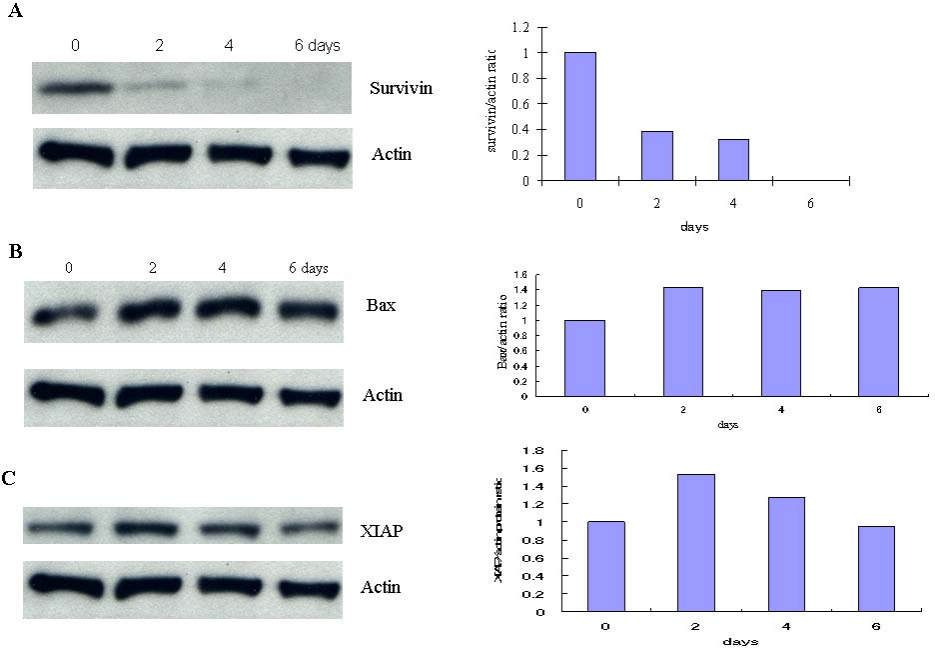
**ATRA affects on the expression of survivin and Bax**. **Panel A **shows the down-regulated expression of survivin after 2-, 4- or 6-day treatment with 180 μM ATRA. **Panel B **shows the up-regulated expression of Bax after 2-, 4- or 6-day treatment with 180 μM ATRA. **Panel C **shows the effect of ATRA on XIAP expression after 2-, 4- or 6-day treatment with 180 μM ATRA.

It is well known that apoptotic process is regulated by many factors. We investigated the expression of inhibitors of apoptosis, survivin, XIAP, and pro-apoptosis Bax. The results showed down-regulation of survivin (Figure 3A) and up-regulation of Bax (Figure 3B). These results were consistent with the appearance of cleaved caspase-3 and PARP in GIST-T1 cells (Figure 2C). However, ATRA did not affect on XIAP expression in GIST-T1 cells by western blot analysis (Figure 3C). All together, the apoptosis induced by ATRA treatment may be regulated at least by down-regulation of survivin and up-regulation of Bax proteins.

### ATRA suppressed the phosphorylation of KIT protein

KIT protein is one of the most important molecules in the pathogenesis of GISTs. Despite clinicopathological difference, most GISTs have a similar genetic profile, gain-of-function mutations of *KIT *or *PDGFRA *[2].

Upon the importance of KIT protein, we examined whether ATRA can suppress KIT activity in GIST-T1 cells. We treated GIST-T1 cells with 180 μM ATRA for the indicated duration. Total cell lysates were subjected to western blot analysis.

**Figure 4:**
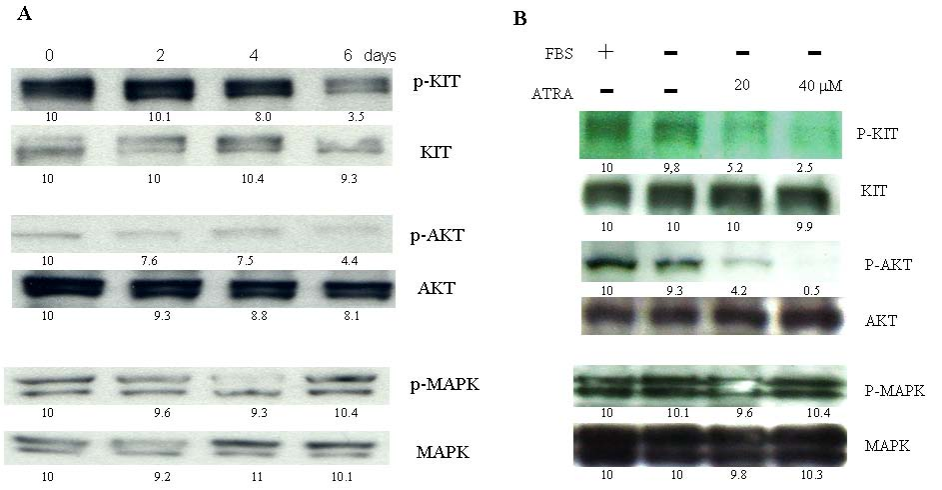
**ATRA suppresses the auto-phosphorylation of KIT and AKT protein but not MAPK activity**. **Panel A **shows the suppression of KIT and AKT activity after 2-, 4- or 6-day treatment with 180 μM ATRA. **Panel B **shows the suppression of KIT and AKT activity after 4 hours treatment with different ATRA concentrations in serum-free media. The results demonstrated that KIT and AKT activity were suppressed by ATRA treatment in a dose- and time-dependent manner but not MAPK activity.

Interestingly, ATRA treatment resulted in suppression of KIT activity after 4-day treatment in GIST-T1 cells (Figure 4A the top row) and GIST-882 cells (data not shown). The suppression of KIT activity in GIST-T1 and GIST-882 cells by ATRA required longer time compared with other reagents such as imatinib or EGCG [25]. In addition, ATRA treatment also suppressed the AKT activity (Figure 4A the middle row) but not MAPK activity (Figure 4A the bottom row) in GIST-T1 cells.

Interestingly, the suppression of KIT and AKT activity by ATRA treatment was enhanced in serum-free media. However, suppression of MAPK activity was not observed even in serum-free media (Figure 4B). The similar results were observed in GIST-882 cells (data not shown).

### ATRA prevented the migration of GIST-T1 cells

Next, to study the migration of GIST-T1 cells *in vitro*, the scratch assay was performed. This method is based on the observation that, upon creation of a new artificial gap, so called a scratch on a confluent cell monolayer, the cell on the edge of the newly created gap will move toward the opening to close the scratch until cell to cell contacts are established again.

**Figure 5:**
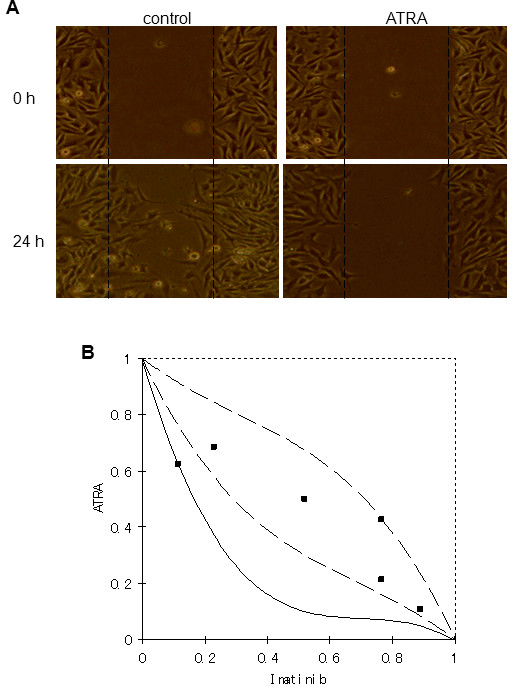
**Panel A shows the result of scratch assay, GIST-T1 cells were treated with or without ATRA (90 μM)**. Migration was observed after 24-hour incubation. **Panel B **shows the isobologram result of drug combination between ATRA and imatinib. This combination resulted in additive effect.

In this study, GIST-T1 cells were seeded with or without ATRA (45, 90 μM) in plates. After 24 hour incubation to get the confluence, a scratch was created. The images of GIST-T1 cells at the beginning and 24 hour later were compared to assess the migration of GIST-T1 cells. The result revealed that 90 μM ATRA inhibited completely migration of GIST-T1 cells compared with the non-ATRA treated dishes (Figure 5A). However, at a lower concentration (45 μM), ATRA inhibited but not completely the migration of these cells (data not shown). All together, the data suggested that ATRA may be useful to prevent the invasion or metastasis of GIST cells.

### Cytotoxic effect of combination with ATRA and imatinib

The result of isobologram was showed in Figure 5B. All data points in the combination fell within the envelope of additivity, the area surrounded by the three lines, suggesting that this combination gave additive effect.

## Discussion

ATRA have been reported to show therapeutic effect on breast and ovarian cancers and APL [28]. However, for the first time we have demonstrated that ATRA suppressed the cell proliferation and induced apoptosis in GIST-T1 cells, suggesting anti-cancer effect of ATRA on GISTs. The cell death inducing mechanism by ATRA in cancers has not yet been fully clarified. In this report we have shown that apoptosis induced by ATRA in GIST-T1 cells are regulated at least by the down-regulation of survivin and up-regulation of Bax (Figure 3A and 3B). Even though XIAP and survivin belong to the same family of apoptotic inhibitors, it is likely that ATRA effected quite differently on expression of XIAP and survivin. Survivin was suppressed in a time dependent manner whereas XIAP was not suppressed by ATRA treatment (Figure 3C). It is likely that survivin may be a target molecule that plays an important role in ATRA-induced apoptosis in GIST-T1 cells. Further studies are definitely necessary for better understanding of the apoptosis-inducing mechanism by ATRA in GIST-T1 cells.

GISTs can be successfully treated with imatinib with the response rate of up to 85% [15,29,30]. However, after a median of 2 years of treatment with imatinib, resistance can develop [15]. The effect of imatinib is mainly due to the suppression of KIT activity. In this study, we found that the suppression of KIT activity (Figure 4A) was also obtained by ATRA treatment. Moreover, we have demonstrated that combination of ATRA and imatinib showed additive effect (Figure 5B) by isobologram, suggesting that the combination of ATRA and imatinib would be a novel therapeutic potential for GISTs. The scratch assay result (Figure 5A) also suggested the useful of ATRA to prevent the invasion or metastasis of GIST cells.

In conclusion, we have demonstrated that ATRA had an ability to inhibit the cell proliferation and migration, inducing apoptosis in GIST-T1 cells. Thus ATRA can have a potential for novel therapeutic agent for GISTs. Since the combination of ATRA and imatinib showed additive effect on GIST-T1 cells, ATRA may be used in combination with imatinib for GISTs treatment.

## Competing interests

The authors declare that they have no competing interests.

## Authors' contributions

HTC and BTKL have carried out the study design, molecular biological work, and statistical analyses and drafted the manuscript. TT has established GIST-T1 cell line. TW and YS have carried out the study design, statistical analyses and drafted the manuscript. All authors read and approved the final manuscript.

## References

[B1] Kindblom LG Remotti HE Aldenborg F Meis-Kindblom JM Gastrointestinal pacemaker cell tumor (GIPACT): gastrointestinal stromal tumors show phenotypic characteristics of the interstitial cells of Cajal Am J Pathol 1998 152 1259 1269 9588894 PMC1858579

[B2] Lasota J Miettinen M Clinical significance of oncogenic KIT and PDGFRA mutations in gastrointestinal stromal tumours Histopathology 2008 53 245 266 10.1111/j.1365-2559.2008.02977.x 18312355

[B3] Hirota S Isozaki K Moriyama Y Hashimoto K Nishida T Ishiguro S Kawano K Hanada M Kurata A Takeda M Gain-of-function mutations of c-kit in human gastrointestinal stromal tumors Science 1998 279 577 580 10.1126/science.279.5350.577 9438854

[B4] Heinrich MC Corless CL Duensing A McGreevey L Chen CJ Joseph N Singer S Griffith DJ Haley A Town A PDGFRA activating mutations in gastrointestinal stromal tumors Science 2003 299 708 710 10.1126/science.1079666 12522257

[B5] Bauer S Hartmann JT de Wit M Lang H Grabellus F Antoch G Niebel W Erhard J Ebeling P Zeth M Resection of residual disease in patients with metastatic gastrointestinal stromal tumors responding to treatment with imatinib Int J Cancer 2005 117 316 325 10.1002/ijc.21164 15900603

[B6] DeMatteo RP Lewis JJ Leung D Mudan SS Woodruff JM Brennan MF Two hundred gastrointestinal stromal tumors: recurrence patterns and prognostic factors for survival Ann Surg 2000 231 51 58 10.1097/00000658-200001000-00008 10636102 PMC1420965

[B7] Buchdunger E Cioffi CL Law N Stover D Ohno-Jones S Druker BJ Lydon NB Abl protein-tyrosine kinase inhibitor STI571 inhibits in vitro signal transduction mediated by c-kit and platelet-derived growth factor receptors J Pharmacol Exp Ther 2000 295 139 145 10991971

[B8] Heinrich MC Griffith DJ Druker BJ Wait CL Ott KA Zigler AJ Inhibition of c-kit receptor tyrosine kinase activity by STI 571, a selective tyrosine kinase inhibitor Blood 2000 96 925 932 10910906

[B9] Okuda K Weisberg E Gilliland DG Griffin JD ARG tyrosine kinase activity is inhibited by STI571 Blood 2001 97 2440 2448 10.1182/blood.V97.8.2440 11290609

[B10] Tuveson DA Willis NA Jacks T Griffin JD Singer S Fletcher CD Fletcher JA Demetri GD STI571 inactivation of the gastrointestinal stromal tumor c-KIT oncoprotein: biological and clinical implications Oncogene 2001 20 5054 5058 10.1038/sj.onc.1204704 11526490

[B11] Dagher R Cohen M Williams G Rothmann M Gobburu J Robbie G Rahman A Chen G Staten A Griebel D Pazdur R Approval summary: imatinib mesylate in the treatment of metastatic and/or unresectable malignant gastrointestinal stromal tumors Clin Cancer Res 2002 8 3034 3038 12374669

[B12] Demetri GD von Mehren M Blanke CD Van den Abbeele AD Eisenberg B Roberts PJ Heinrich MC Tuveson DA Singer S Janicek M Efficacy and safety of imatinib mesylate in advanced gastrointestinal stromal tumors N Engl J Med 2002 347 472 480 10.1056/NEJMoa020461 12181401

[B13] Heinrich MC Corless CL Blanke CD Demetri GD Joensuu H Roberts PJ Eisenberg BL von Mehren M Fletcher CD Sandau K Molecular correlates of imatinib resistance in gastrointestinal stromal tumors J Clin Oncol 2006 24 4764 4774 10.1200/JCO.2006.06.2265 16954519

[B14] Koyama T Nimura H Kobayashi K Marushima H Odaira H Kashimura H Mitsumori N Yanaga K Recurrent gastrointestinal stromal tumor (GIST) of the stomach associated with a novel c-kit mutation after imatinib treatment Gastric Cancer 2006 9 235 239 10.1007/s10120-006-0368-5 16952044

[B15] Verweij J Casali PG Zalcberg J LeCesne A Reichardt P Blay JY Issels R van Oosterom A Hogendoorn PC Van Glabbeke M Progression-free survival in gastrointestinal stromal tumours with high-dose imatinib: randomised trial Lancet 2004 364 1127 1134 10.1016/S0140-6736(04)17098-0 15451219

[B16] Demetri GD van Oosterom AT Garrett CR Blackstein ME Shah MH Verweij J McArthur G Judson IR Heinrich MC Morgan JA Efficacy and safety of sunitinib in patients with advanced gastrointestinal stromal tumour after failure of imatinib: a randomised controlled trial Lancet 2006 368 1329 1338 10.1016/S0140-6736(06)69446-4 17046465

[B17] Shah NP Tran C Lee FY Chen P Norris D Sawyers CL Overriding imatinib resistance with a novel ABL kinase inhibitor Science 2004 305 399 401 10.1126/science.1099480 15256671

[B18] Debiec-Rychter M Cools J Dumez H Sciot R Stul M Mentens N Vranckx H Wasag B Prenen H Roesel J Mechanisms of resistance to imatinib mesylate in gastrointestinal stromal tumors and activity of the PKC412 inhibitor against imatinib-resistant mutants Gastroenterology 2005 128 270 279 10.1053/j.gastro.2004.11.020 15685537

[B19] Collins MD Mao GE Teratology of retinoids Annu Rev Pharmacol Toxicol 1999 39 399 430 10.1146/annurev.pharmtox.39.1.399 10331090

[B20] Morriss-Kay GM Ward SJ Retinoids and mammalian development Int Rev Cytol 1999 188 73 131 full_text 10208011 10.1016/s0074-7696(08)61566-1

[B21] Kastner P Mark M Chambon P Nonsteroid nuclear receptors: what are genetic studies telling us about their role in real life? Cell 1995 83 859 869 10.1016/0092-8674(95)90202-3 8521510

[B22] Napoli JL Biochemical pathways of retinoid transport, metabolism, and signal transduction Clin Immunol Immunopathol 1996 80 S52 62 10.1006/clin.1996.0142 8811064

[B23] Bastien J Rochette-Egly C Nuclear retinoid receptors and the transcription of retinoid-target genes Gene 2004 328 1 16 10.1016/j.gene.2003.12.005 15019979

[B24] Taguchi T Sonobe H Toyonaga S Yamasaki I Shuin T Takano A Araki K Akimaru K Yuri K Conventional and molecular cytogenetic characterization of a new human cell line, GIST-T1, established from gastrointestinal stromal tumor Lab Invest 2002 82 663 665 10.1038/labinvest.3780461 12004007

[B25] Chi HT Vu HA Iwasaki R Thao le B Hara Y Taguchi T Watanabe T Sato Y Green tea (-)-epigalocatechin-3-gallate inhibits KIT activity and causes caspase-dependent cell death in gastrointestinal stromal tumor including imatinib-resistant cells Cancer Biol Ther 2009 8 1934 1939 19770581 10.4161/cbt.8.20.9594

[B26] Steel GG Peckham MJ Exploitable mechanisms in combined radiotherapy-chemotherapy: the concept of additivity Int J Radiat Oncol Biol Phys 1979 5 85 91 422420 10.1016/0360-3016(79)90044-0

[B27] Kroemer G Galluzzi L Vandenabeele P Abrams J Alnemri ES Baehrecke EH Blagosklonny MV El-Deiry WS Golstein P Green DR Classification of cell death: recommendations of the Nomenclature Committee on Cell Death 2009 Cell Death Differ 2009 16 3 11 10.1038/cdd.2008.150 18846107 PMC2744427

[B28] Fields AL Soprano DR Soprano KJ Retinoids in biological control and cancer J Cell Biochem 2007 102 886 898 10.1002/jcb.21530 17902161

[B29] van Oosterom AT Judson IR Verweij J Stroobants S Dumez H Donato di Paola E Sciot R Van Glabbeke M Dimitrijevic S Nielsen OS Update of phase I study of imatinib (STI571) in advanced soft tissue sarcomas and gastrointestinal stromal tumors: a report of the EORTC Soft Tissue and Bone Sarcoma Group Eur J Cancer 2002 38 Suppl 5 S83 87 10.1016/S0959-8049(02)80608-6 12528778

[B30] Blanke CD Rankin C Demetri GD Ryan CW von Mehren M Benjamin RS Raymond AK Bramwell VH Baker LH Maki RG Phase III randomized, intergroup trial assessing imatinib mesylate at two dose levels in patients with unresectable or metastatic gastrointestinal stromal tumors expressing the kit receptor tyrosine kinase: S0033 J Clin Oncol 2008 26 626 632 10.1200/JCO.2007.13.4452 18235122

